# Determinants of Secondary Hyperparathyroidism in Bariatric Patients after Roux-en-Y Gastric Bypass or Sleeve Gastrectomy: A Pilot Study

**DOI:** 10.1155/2015/984935

**Published:** 2015-04-09

**Authors:** Andreas Alexandrou, Evangelia Tsoka, Eleni Armeni, Demetrios Rizos, Theodoros Diamantis, Areti Augoulea, Constantinos Panoulis, Theodoros Liakakos, Irene Lambrinoudaki

**Affiliations:** ^1^1st Department of Surgery, Laiko Athens General Hospital, University of Athens, 17 Agiou Thoma Street, 11527 Athens, Greece; ^2^2nd Department of Obstetrics and Gynecology, Aretaieio Hospital, University of Athens, 76 Vas. Sofias Avenue, 11528 Athens, Greece; ^3^Hormonal Laboratory, Aretaieio Hospital, University of Athens, 76 Vas. Sofias Avenue, 11528 Athens, Greece

## Abstract

*Objective*. Nutritional deficiencies are common after bariatric surgery. We aimed to assess the prevalence and possible predictors of secondary hyperparathyroidism (SHPT) in bariatric patients. *Methods*. A total of 95 patients who had undergone Roux-en-Y gastric bypass (RYGB) or sleeve gastrectomy (SG) were assessed after a median of 3 years after surgery. Anthropometric/demographic and weight-loss parameters were compared according to the presence of SHPT, independently for men/premenopausal women and postmenopausal women. *Results*. SHPT was highly prevalent (men/premenopausal women, 52.1%; postmenopausal women, 31.9%). Among men/premenopausal women, multivariate analysis indicated that SHPT was predicted by (a) 25-hydroxyvitamin D levels (Exp(*B*) = 0.869, *P*-value = 0.037), independently of age, sex, smoking; (b) calcium (Exp(*B*) = 0.159, *P*-value = 0.033) and smoking, independently of age and sex; (c) magnesium (Exp(*B*) = 0.026, *P*-value = 0.046) and smoking, independently of age and sex. Among postmenopausal women, SHPT was predicted by menopausal age independently of age, smoking, and levels of 25-hydroxyvitamin D or calcium. The development of SHPT was not associated with the type of surgery. *Conclusions*. RYGB and SG exhibited similar effects regarding the regulation of the hypothalamus-pituitary-parathyroid axis after surgery. Vitamin D status and menopausal age appear to determine SHPT on the long term. SHPT should be sought and vigorously treated with calcium and vitamin D supplementation.

## 1. Introduction

Bariatric surgery is one of the most effective treatments of morbid obesity, resulting in significant weight loss when compared with a more conservative approach [1, 2]. Furthermore, bariatric operations seem to decrease the impact of obesity-induced comorbidities, like diabetes mellitus, cardiovascular disease, arterial hypertension, dyslipidemia, and chronic anovulation, while they have been related to an overall improvement of the health-related quality of life [1, 3].

Among the various bariatric procedures, Roux-en-Y Gastric Bypass (RYGB) represents the gold-standard method, while sleeve gastrectomy (SG) is becoming increasingly popular worldwide, as a stand-alone procedure [1, 4, 5]. RYGB is a combined operation, where part of the stomach along with the duodenum and a large part of the jejunum are bypassed, resulting in both food restriction and malabsorption, thus producing a significant excess weight loss. On the other hand, malabsorption of significant micronutrients like calcium and vitamin D represents a common clinical issue in the long run [4, 6]. SG is a novel, restrictive approach, during which the majority of the fundus and the major curvature of the stomach are removed, leading to a quick fullness sensation [5]. As we previously reported, both types of bariatric operations result in significant anatomical and functional alterations of the gastrointestinal tract, leading to a lifetime risk of vitamin and micronutrient deficiencies [7].

The integrity of the gastrointestinal tract bears a significant role in calcium homeostasis, an interplay mainly regulated by vitamin D and parathormone (PTH) mediated calcium absorption [8]. Secondary hyperparathyroidism occurs frequently after bariatric surgery; however, the net effect of bariatric procedures on the absorption of calcium and vitamin D is not clearly defined [1, 9]. Existing studies assessed mainly mixed populations of operated morbidly obese patients, so that the net effect of weight-loss surgery on calcium metabolism in men and premenopausal or postmenopausal women has not yet been clearly evaluated, apart from one exception [10].

Given the significant impact of the gastrointestinal tract in the regulation of the hypothalamus-pituitary-parathyroid (HPP) axis, the present study aimed to investigate the long-term prevalence of SHPT as well as possible risk factors, in a sample of morbidly obese Greek patients, according to the reproductive status, as well as the type of bariatric surgery performed, namely, RYGB or SG.

## 2. Materials and Methods

This pilot study included patients who had undergone RYGB or SG surgery in our department from 2000 until 2011. This department serves as a referral center for bariatric patients from Central and Southern Greece. Patients who undergo bariatric surgery return to their permanent residence, with recommendations for adherence to multivitamin supplementation and for at least two follow-up visits to their local GP. From the 226 patients operated during the aforementioned period, 211 patients had a complete data file and were eligible for inclusion in the study. Among the 165 patients, who were located by phone, 95 patients consented to participate in the study (response rate 57.6%). Patients who refused to participate (*N* = 70) where mainly residents from Southern Greece, who were not able to travel to Athens for the purpose of the study, mentioning economic problems as well as time limitations. The total of 46 patients who could not be located by telephone included 25 patients who actually responded positively to the telephonic invitation but never appeared in the clinic for unknown reasons, as well as 21 patients who never answered the phone. All patients signed an informed consent and the study was approved by the Ethics Committee of Aretaieion Hospital, University of Athens.

Eligibility criteria for surgery were [1] a body mass index (BMI) ≥ 40 kg/m^2^ or ≥35 kg/m^2^ in patients with obesity-related comorbidities, failure of previous attempts at weight reduction, and expected adherence to postoperative care (e.g., follow-up visits and use of dietary supplements). Exclusion criteria were (i) reversible endocrine or other disorders causing obesity, (ii) preoperative gut malabsorption syndrome, (iii) gastric-kidney-liver disease, (iv) preoperative intake of medication affecting bone metabolism (e.g., calcium and vitamin D supplements, glucocorticoids, diuretics, and antiepileptic drugs), (v) uncontrolled or severe psychiatric illness, and (vi) current drug or alcohol abuse.

Surgical techniques were standardized and remained the same throughout the whole study period. RYGB was performed by constructing a gastric pouch of approximately 30 mL with the use of the Echelon 60 mm linear stapler (Ethicon). The alimentary limb had a length of 150 cm, and the respective length of the biliopancreatic limb was always 100 cm. The gastrojejunal anastomosis was constructed in a semimanual way. The posterior wall was constructed with the use of the linear stapler (EndoGIA 45 mm, Ethicon) while the anterior wall was sutured manually. The width of the anastomosis was approximately 1 cm. The jejunojejunal anastomosis was constructed with the use of the Echelon 60 mm linear stapler. All dissections were performed with the use of the Harmonic scalpel (Ethicon). On the other hand, for the construction of the gastric sleeve, the harmonic scalpel and the Echelon linear stapler 60 mm (both Ethicon) were used, while the calibration of the gastric sleeve was performed with the use of the 9.1 mm endoscope as we have previously reported [5].

Postoperatively, all patients were advised to follow a high calcium diet, as well as to take supplemental multivitamins (two tablets daily), chewable tablets of 500 mg calcium citrate (500 mg, twice daily), and vitamin D_3_ (400 IU, twice daily). We advised our patients to consume commercially available multivitamin products (Centrum multivitamins), which included vitamin B12 2.5 *μ*g (100% RDA), vitamin D 5 *μ*g (100% RDA), folic acid 200 *μ*g (100% RDA), calcium 162 mg (20% RDA), phosphorus 125 mg (18% RDA), magnesium 100 mg (27% RDA), and Fe 5 mg (36% RDA). Moreover, patients were asked to present to their local GP at 6 and 12 months for follow-up evaluation within the first postsurgical year, while subjects diagnosed with vitamin D deficiency were advised to take 5000 IU of oral vitamin D daily and to be reassessed 3 months later. The local GP of each patient was responsible for assessing multivitamin intake, using pill counts at a 6-month interval.

Anthropometric data and a detailed medical history were recorded for every subject. Demographic, lifestyle parameters, and medication history were obtained using a questionnaire composed for the evaluation of demographic and lifestyle parameters of the recruited patients. (see Supplement 1 in Supplementary Material available online at http://dx.doi.org/10.1155/2015/984935). Data on preoperative weight and on excess weight loss rate were derived from patient files; however, no preoperative biochemical values were available. Blood pressure, waist to hip ratio (WHR), weight, and height were assessed in the morning in light clothing. BMI was calculated using the equation BMI = weight (kg)/height^2^ (m^2^). Patients were instructed to abstain from food and smoking for 12 hours, and subsequently fasting venous blood samples were drawn for biochemical evaluation at 8.30–9.30 am. The menopausal status was defined as 12 consecutive months without menses. Weight loss was expressed as percentage (%) of excess weight loss (%EWL). Excess weight was estimated using the middle of the Metropolitan Life Insurance tables [11]. Excess BMI was defined as the difference between the BMI value of each individual patient at the follow up visit minus the cut-off value 25 kg/m^2^. According to serum parathormone (PTH) levels, subjects were divided into two subsamples: (1) subjects with secondary hyperparathyroidism (SHPT, defined as serum PTH above the high normal limit: 62 pg/mL) and (2) subjects with normal levels of PTH [12]. Vitamin D deficiency was defined as serum levels ≤10 ng/mL, while serum levels of vitamin D ≤20 ng/mL were defined as suboptimal [13]. Folate deficiency was defined as serum folate concentration <4 ng/mL [14].

### 2.1. Biochemical Assays

Serum glucose, total cholesterol, triglycerides, and high density lipoprotein-cholesterol (HDL-C) were assessed enzymatically by an autoanalyzer (ARCHITECT-ci8200, Abbott Diagnostics Laboratories, Abbott Park, IL 60064, USA, and Abbott, 65205 Wiesbaden, Germany). Creatinine levels were determined by an enzymatic method. Serum levels of total calcium, magnesium, phosphorus, and folate, as well as 25-hydroxyvitamin D and PTH were assessed enzymatically by an autoanalyzer (ARCHITECT ci4100 Integrated System, Abbott Diagnostics Laboratories, Abbott Park, IL 60064, USA, and Abbott, 65205 Wiesbaden, Germany). LDL-cholesterol (LDL-C) was estimated using the Friedewald equation (LDL-C = total cholesterol − triglycerides/5 − HDL-C).

### 2.2. Statistical Analysis

Data analysis was performed using SPSS version 17.0 (Statistical Package for Social Sciences, Chicago, IL, USA) independently for men/premenopausal women as well as for postmenopausal women. Data on qualitative characteristics are expressed as percent values, while data on quantitative characteristics are expressed as means ± Standard Deviation (SD). Differences between subjects with SHPT and the rest of the sample, serving as controls, were assessed using analysis of variance (ANOVA) or the Mann-Whitney *U* test for continuous variables and Chi-square (*χ* ^2^) analysis for categorical variables. Logarithmic transformation was used in cases of skewed data. Stepwise multivariate logistic regression analysis was used to identify significant determinants of SHPT, adjusting for various SHPT-related risk factors. Statistical significance was set at the 0.05 level.

## 3. Results

Table 1 presents the mean values of demographic and anthropometric characteristics of the sample at recruitment, according to the type of surgery. The median length of postop follow-up was 3 years.

Table 2 presents the results of the comparison of main anthropometric and biochemical parameters according to the presence of SHPT, independently for men/premenopausal women (*N* = 48) as well as postmenopausal women (*N* = 47). SHPT was highly prevalent in the subgroup of men/premenopausal women, in up to 52.1% (25/48) of patients. Levels of 25(OH) vitamin D differed significantly between patients with SHPT and controls (9.44 ± 4.20 ng/dL versus 12.76 ± 5.62 ng/dL, *P*-value = 0.028). Moreover we observed lower levels of magnesium and calcium in patients with SHPT when compared with controls who presented with higher levels (SHPT versus controls, magnesium: 2.19 ± 0.23 mg/dL versus 2.38 ± 0.29 mg/dL, *P*-value = 0.036; calcium: 8.98 ± 0.54 mg/dL versus 9.30 ± 0.39 mg/dL, *P*-value = 0.042). However, magnesium levels were found within the normal range in the subsample of men/premenopausal women. Finally, the presence of suboptimal levels of vitamin D and vitamin D deficiency tended to associate with the presence of SHPT (*P*-value 0.055 and 0.094, resp.). Current smoking exhibited a borderline protective effect on the development of SHPT (*P*-value = 0.051), whereas the type of surgery was not associated with the mobilization of the HPP axis. No other significant differences were observed postoperatively between patients with SHPT and controls. With respect to the subgroup of postmenopausal women, SHPT was prevalent in up to 31.9% (15.47) of cases. SHPT was associated with vitamin D deficiency (SHPT versus controls: 78.6% versus 40.7%, *P*-value = 0.021); levels of vitamin D, however, could not exhibit a significant effect on the mobilization of the HPP axis. Levels of magnesium were normal in the majority of subjects, with deficiency being observed only in up to 3.8% of postmenopausal women. In addition, menopausal age exhibited a borderline effect on the development of SHPT (SHPT versus controls: 8.14 ± 6.40 versus 3.96 ± 5.14, *P*-value = 0.068, MW *U* test). No other significant differences were observed between postmenopausal women SHPT and controls.

Models of stepwise multiple regression analysis were used, aiming to identify potential predictors of SHPT, and the results are presented in Table 3. With respect to the subgroup of men and premenopausal women, the development of SHPT was predicted by (a) the levels of 25(OH) vitamin D (Exp(*B*) = 0.869, *P*-value = 0.037) in a model that included age, sex, and current smoking; (b) serum levels of calcium (Exp(*B*) = 0.159, *P*-value = 0.033) and current smoking (Exp(*B*) = 6.966, *P*-value = 0.013); as well as (c) serum levels of magnesium (Exp(*B*) = 0.026, *P*-value = 0.046) and current smoking (Exp(*B*) = 5.202, *P*-value = 0.033), with the two latter models including also age and sex as potential confounders. With respect to the subgroup of postmenopausal women, we observed an interesting direct effect of menopausal age on the development of SHPT, irrespective of age, current smoking, and levels of 25(OH) vitamin D or serum levels of calcium.

## 4. Discussion

The present study demonstrates the high frequency of secondary hyperparathyroidism and vitamin D deficiency on the long term after bariatric surgery, a finding observed both in men/premenopausal women and in postmenopausal women. The analysis has been performed independently for men/premenopausal women and for postmenopausal women, aiming to evaluate the confounding role of menopause on bone metabolism and calcium homeostasis. Interestingly, the type of surgery had no effect on the development of these nutrient disorders. On the contrary, the development of SHPT was mainly determined by the vitamin D status as well as smoking with respect to men and premenopausal women. Menopausal age was the main determinant of SHPT for postmenopausal women.

Bariatric operations have been associated with significant alterations in the HPP axis with vitamin D deficiency and SHPT representing major problems after surgery [9]. According to recent evidence, rates of hypovitaminosis D in patients who had undergone bariatric procedures range from 33% up to 96.7%, in agreement with our results [9]. In addition, SHPT is reported frequently after bariatric operations, while elevated serum levels of PTH have been identified in up to 53% of patients [15]. Obese individuals seem to be more vulnerable regarding the development of vitamin D deficiency, mainly due to sunlight avoidance, relative inactivity, reduced bioavailability of the fat-soluble vitamin, and inhibited hepatic vitamin activation [9, 15, 16]. The extent of developing disorders of the HPP axis can be partially explained by the differences in preoperative vitamin D status, differences in latitude, season of data collection, differing definitions of nutrient and vitamin deficiencies, variable adherence, different doses of supplementation after surgery, and loss of follow-up after surgery [9, 15, 16].

According to our findings, fluctuation in serum levels of PTH is mainly determined by levels of circulating 25(OH) vitamin D as well as by the extent of weight loss after surgery. Reduced serum levels of vitamin D and subsequent limited calcium absorption induce the development of SHPT, whereas increased PTH production results in the mobilization of the skeletal calcium, aiming to preserve normocalcemia [9, 15]. The majority of data supports an inverse association between PTH and levels of vitamin D [16–18], in agreement with our results. Interestingly, a recent study evaluated a large sample of 293 cases who had undergone RYGB surgery with a median follow-up of 11 ± 2.8 years, aiming to identify predictors of vitamin D deficiency [19]. This study reported significant correlations between serum levels of PTH and circulating vitamin D (*P*-value < 0.001) [19].

Moreover, the postoperative levels of PTH were predicted by the rate of weight loss after surgery, whereas the preoperative BMI had a mild effect on postsurgical PTH levels, an effect that was further attenuated after adjustments for other potential confounders. Levels of PTH after bariatric surgery have been negatively associated with preoperative BMI in a case-control study of 48 female patients and 50 age- and race-matched controls [18]. Moreover, postoperative weight loss has been negatively associated with levels of PTH [20] and positively with levels of vitamin D after surgery [21]. Interestingly, Lin et al. [22] observed significant correlations between adipose tissue mass and plasma levels of 25(OH) vitamin D, further supporting our findings. This study [22] suggested an increased storage of vitamin D in patients with excessive adipose tissue, with a concomitant release during the stage of initial weight loss.

The type of surgery was not associated with serum levels of vitamin D or with the prevalence of SHPT according to our findings. Moizé et al. [23] reported that both types of surgery were associated with similarly high rates of vitamin D deficiency 5 years after surgery (44% for SG and 51% for RYGB). Furthermore, Kehagias et al. [24] reported no difference in the rate of SHPT between SG and RYGB patients 3 years after surgery. The opposite results were observed by Gehrer et al. [25], who showed that vitamin D deficiency was more frequent in the RYGB patients (52%) compared to those who underwent SG (32%). The same was observed in the frequency of SHPT, after an average duration of 2 years after surgery (33% in RYGB versus 14% in SG) [25]. In a recent prospective trial in 100 patients who were randomly assigned either to RYGB or to SG, SHPT was evident in 15% of RYGB patients and in no SG patient 6 months after surgery [26]. It should be noted, however, that patients were supplemented with high doses of parenteral vitamin D [26], in contrast to our patients who exhibited very low compliance in the prescribed supplement intake. Coupaye et al. [27] aimed to compare the two operations assessing nutritional deficiencies in the first year after surgery in patients who were matched for age, gender, and postoperative weight. At 12 months, although the levels of serum PTH were not different between the two groups, RYGB patients had lower circulating 25(OH) vitamin D compared to SG [27].

The extent to which bariatric operations induce malabsorption of vitamin D and subsequently the development of SHPT remains unknown for a number of reasons. The effect of induced weight loss on serum levels of parathormone is determined by surgical and nonsurgical factors. Decreased postoperative gastric acid secretion is observed mainly in operations with a malabsorptive component, leading to impaired intestinal calcium absorption [8]. In addition, the extent of fat malabsorption may affect the absorption of fat soluble vitamins like vitamin D [6]. In addition, patients who underwent bariatric operations report limited intake of dairy products due to dietary intolerance after surgery [9]. Interestingly, nonsurgical factors like smoking and menopausal age might also affect the mobilization of the HPP axis, further complicating this association, according to our findings. Representing a known risk factor of bone loss, smoking may interact with 25 hydroxylase in the liver, resulting in lower levels of 25(OH) vitamin D [28]. Menopausal age has been independently associated with reduced levels of vitamin D, further deteriorating the effect of weight loss on the mobilization of the HPP axis [29, 30]. However, serum levels of calcium should not be regarded as an accurate indicator of calcium absorption, since mobilization of the HPP axis results in further release of calcium from bone, which is subsequently utilized to maintain plasma levels of calcium [6, 15]. Finally, the preoperative status of micronutrient deficiencies further determines the extent to which bariatric procedures induce increased PTH production [9, 16].

Limitations of this study include its cross-sectional design, prohibiting the demonstration of prospective cause-effect associations. Adherence to vitamin or mineral supplements was estimated indirectly, based on patient information. Furthermore, the absence of presurgery data did not allow assessing the net effect of each operation on the development of SHPT. Moreover, no data were available regarding the extent of sun exposure of the patients included in the present study. Finally, the sample size is relatively small so that our findings can be extrapolated to the general bariatric population only with caution.

Concluding, SHPT is highly prevalent after SG and RYGB in the long term. The type of surgery was not associated with either vitamin D or PTH levels. On the contrary, the only significant parameters affecting SHPT were circulating vitamin D and the degree of weight loss. Calcium metabolism should be closely monitored in bariatric patients after surgery and any deficits detected should be vigorously treated.

## Supplementary Material

The Supplemental file refers to the questionnaire, which was composed in order to evaluate the demographic and lifestyle parameters of the participants.

## Figures and Tables

**Table 1:** Mean values of demographic, anthropometric, and biochemical characteristics of the sample at recruitment according to the type of surgery.

Variable	Mean ± SD or frequency (%)		ANOVA or *χ* ^2^
	RYGB *N* = 55	SG *N* = 40	*P* value
Age (years)	44.8 ± 9.9	43.4 ± 11.2	0.509
Gender (female sex)	**72.7%**	**90.0%**	**0.038**
Alcohol consumption	3.64%	2.50%	0.755
Current smoking	50.9%	47.5%	0.743
SBP (mmHg)	125.7 ± 21.5	128.9 ± 21.1	0.496
DBP (mmHg)	82.6 ± 11.6	81.4 ± 11.9	0.627
BMI before surgery (kg/m^2^)	49.1 ± 6.1	51.5 ± 7.2	0.084

SBP: systolic blood pressure; DBP: diastolic blood pressure; BMI: body mass index.

Statistical significance was set at the level of *P* value <0.05.

**Table 2:** Comparison of main anthropometric and biochemical parameters according to the presence of secondary hyperparathyroidism after surgery, independently for men/premenopausal women and for postmenopausal women.

	Mean ± SD or frequency (%)		ANOVA or *χ* ^2^
	SHPT	Controls	*P* value
Men and premenopausal women (*N* = 48)			
Age (years)	40.7 ± 7.8	43.3 ± 40.2	0.335
BMI (kg/m^2^)	34.7 ± 6.1	33.5 ± 5.4	0.484
WHR	0.83 ± 0.21	0.86 ± 0.10	0.544
Excess weight loss (%)	57.7 ± 16.5	59.9 ± 15.1	0.654
Duration after surgery (years)	5.36 ± 3.40	4.38 ± 2.56	0.292
Parathormone (pg/dL)	82.8 ± 22.0	47.7 ± 11.4	**<0.001**
25(OH)VitD (ng/dL)	9.44 ± 4.20	12.76 ± 5.62	**0.028**
Log-creatinine (mg/dL)	0.69 ± 1.28	0.72 ± 1.20	0.508
Calcium (mg/dL)	8.98 ± 0.54	9.30 ± 0.39	**0.042**
Magnesium (mg/dL)	2.19 ± 0.23	2.38 ± 0.29	**0.036**
Phosphorus (mg/dL)	3.39 ± 0.37	3.47 ± 0.48	0.592
Folate deficiency (<4 ng/mL, %)	23.1%	40%	0.339
Supplemental calcium and/or vitamin D (%)	4.17%	4.76%	0.923
Suboptimal vitamin D	100%	85.7%	0.055
Vitamin D deficiency	58.3%	33.3%	0.094
Current smoking	37.5%	66.7%	0.051
Type of surgery: sleeve gastrectomy^1^	22.7%	28.6%	0.661
Postmenopausal women (*N* = 47)			
Secondary hyperparathyroidism			
Age (years)	51.6 ± 11.6	46.7 ± 10.0	0.166
Menopausal age (years)	8.14 ± 6.40	3.96 ± 5.14	0.068^#^
BMI (kg/m^2^)	34.4 ± 5.8	32.9 ± 7.2	0.519
WHR	0.85 ± 0.06	0.81 ± 0.08	0.152
Excess weight loss (%)	52.9 ± 13.1	62.3 ± 21.7	0.163
Log-duration after surgery (years)	3.50 ± 1.50	2.99 ± 1.72	0.343
Parathormone (pg/dL)	94.5 ± 32.8	49.6 ± 7.3	**<0.001**
25(OH)VitD (ng/dL)	9.34 ± 7.82	12.88 ± 8.95	0.219
Creatinine (mg/dL)	0.71 ± 0.14	0.69 ± 0.18	0.734
Calcium (mg/dL)	9.40 ± 0.80	9.29 ± 0.41	0.641
Magnesium (mg/dL)	2.15 ± 0.30	2.16 ± 0.20	0.977
Phosphorus (mg/dL)	3.67 ± 0.36	3.64 ± 0.62	0.912
Folate deficiency (<4 ng/mL, %)	16.7%	36.3%	0.394
Supplemental calcium and/or vitamin D (%)	21.4%	7.4%	0.193
Suboptimal vitamin D	92.9%	92.6%	0.975
Vitamin D deficiency	**78.6%**	**40.7%**	**0.021**
Current smoking	28.6%	55.6%	0.100
Type of surgery: sleeve gastrectomy^1^	61.5%	40.7%	0.217

SHPT: secondary hyperparathyroidism; BMI: body mass index; WHR: waist to hip ratio; 25(OH)VitD: 25-hydroxyvitamin D. ^1^Reference category: Roux-en-Y gastric bypass; statistical significance was set at the level of *P* value <0.05. ^#^Mann-Whitney *U* test was used due to insufficient normality of data.

**Table 3:** Stepwise multivariate logistic regression analysis with the presence of secondary hyperparathyroidism as dependent variable and potential risk factors as independent variables.

Secondary hyperparathyroidism	Model *χ* ^2^	Exp(*B*)	*P* value
Men and premenopausal women			
Model 1			
Age (years)	5.005	0.951	0.329
Sex^1^		0.269	0.604
Current smoking		2.362	0.124
25-hydroxyvitamin D (ng/mL)		**0.869**	**0.037**
Model 2			
Age (years)	11.524	0.148	0.701
Sex^1^		0.612	0.434
Current smoking		**6.966**	**0.013**
Calcium (mg/dL)		**0.159**	**0.033**
Model 3			
Age (years)	10.082	1.753	0.186
Sex^1^		1.096	0.295
Current smoking		**5.202**	**0.033**
Magnesium (mg/dL)		**0.026**	**0.046**
Postmenopausal women			
Model 1			
Age (years)	4.689	0.002	0.967
YSM (years)		**1.132**	**0.038**
Current smoking		0.549	0.459
25-hydroxyvitamin D (ng/mL)		1.808	0.179
Model 2			
Age (years)	5.879	0.840	0.359
YSM (years)		**1.195**	**0.025**
Current smoking		0.414	0.520
Calcium (mg/dL)		0.023	0.878
Model 3			
Age (years)	3.745	0.992	0.907
YSM (years)		1.256	0.110
Current smoking		3.240	0.383
Magnesium (mg/dL)		1.876	0.768

YSM: years since menopause.

^1^Reference category: male sex.

Statistical significance was set at the level of *P* value <0.05.
