# Supplementary material for: Determinants of Secondary Hyperparathyroidism in Bariatric Patients after Roux-en-Y Gastric Bypass or Sleeve Gastrectomy: A Pilot Study
Source: Int J Endocrinol. 2015 Apr 9;2015:984935. doi: 10.1155/2015/984935 (PMC4408644; doi:10.1155/2015/984935)
Supplement: Supplementary file 1 — The Supplemental file refers to the questionnaire, which was composed in order to evaluate the demographic and lifestyle parameters of the participants. [file 984935.f1.pdf]

Questionnaire

Evaluation of demographic and lifestyle parameters.

1. Race
   (0: Caucasian, 1: Non-Caucasian)

2. Ethnicity
   (0: Greek, 1: Other)

3. Type of surgery
   (0: Roux-En-Y-Gastric Bypass, 1:Sleeve Gastrectomy)

4. Gender (0: male, 1: female)

5. Date of birth

6. Marital status
   (0: married, 1: single/divorced/widowed)

7. Highest degree obtained
   (0: no education, 1: primary education, 2: secondary education, 3: university degree)

8. Physical exercise
   (0: not at all, 1: ≤ 1 hour weekly, 2: 1 – 4 hours weekly, 3: > 3 hours per week)

9. Alcohol consumption
   (0: never, 1: occasionally, 2: daily)

10. Smoking habits
    (0: never or past smoker, 1: current smoker)

11. Medical history:
    (0: no, 1: yes)
    - Diabetes mellitus?
    - Hypertension?
    - Coronary heart disease?
    - Dyslipidaemia

- Arterial thrombosis
- Thromboembolism
- Thyroid dysfunction
- Other (specify)

12. Medication administration (0: no, 1: yes)
    - Oral hypoglycemic drugs (metformin, sulfonylureas, a-glycosidase inhibitors, DPP4, GLP-1)
    - Insulin (glargine, levemir or human isophane insulin)
    - Angiotensin II receptor antagonist
    - Calcium channel blocker
    - Diuretic
    - B-Blocker
    - Digoxin
    - Hypolipidemic drugs: statins, fibrates, nicotinic acid
    - Acenocoumarol
    - Heparin
    - Levothyroxin
    - Antithyroid drugs: carbimazole, thiamazole
    - NSAIDs

13. vitamin and micronutrient supplementation (0: no, 1: yes)
    - Calcium and vitamin D
    - Ferrum
    - Follic acid
    - Vitamin B12
    - Multivitamins

14. Menopausal status (for women)
    (0: premenopausal, 1: postmenopausal)

15. For women: Years since menopause
